# Clinical use of Convalescent Plasma in the COVID‐19 pandemic: a transfusion‐focussed gap analysis with recommendations for future research priorities

**DOI:** 10.1111/vox.12973

**Published:** 2020-09-03

**Authors:** Arwa Z. Al‐Riyami, Richard Schäfer, Karin van den Berg, Evan M. Bloch, Lise J. Estcourt, Ruchika Goel, Salwa Hindawi, Cassandra D. Josephson, Kevin Land, Zoe K. McQuilten, Steven L. Spitalnik, Erica M. Wood, Dana V. Devine, Cynthia So‐Osman

**Affiliations:** ^1^ Department of Haematology Sultan Qaboos University Hospital Muscat Sultanate of Oman; ^2^ Institute for Transfusion Medicine and Immunohaematology German Red Cross Blood Donor Service Baden‐Württemberg‐Hessen gGmbH Goethe University Hospital Frankfurt am Main Germany; ^3^ Medical Division Translational Research Department South African National Blood Service Port Elizabeth South Africa; ^4^ Division Clinical Haematology Department of Medicine University of Cape Town Cape Town South Africa; ^5^ Department of Pathology Johns Hopkins University School of Medicine Baltimore MD USA; ^6^ Radcliffe Department of Medicine University of Oxford and NHS Blood and Transplant Oxford UK; ^7^ Division of Transfusion Medicine Department of Pathology Johns Hopkins Hospital Baltimore MD USA; ^8^ Division of Hematology/Oncology Simmons Cancer Institute at SIU School of Medicine and Mississippi Valley Regional Blood Center Springfield IL USA; ^9^ Haematology Department Faculty of Medicine King Abdulaziz University Jeddah Saudi Arabia; ^10^ Department of Pathology and Laboratory Medicine Center for Transfusion and Cellular Therapies Emory University School of Medicine Atlanta GE USA; ^11^ Department of Pediatrics Aflac Cancer Center and Blood Disorders Children's Healthcare of Atlanta Emory University School of Medicine Atlanta GE USA; ^12^ Corporate Medical Affairs Vitalant Phoenix AZ USA; ^13^ Department of Pathology UT Health Science San Antonio San Antonio TX USA; ^14^ Transfusion Research Unit School of Public Health and Preventive Medicine Monash University Melbourne VIC Australia; ^15^ Department of Clinical Haematology Monash Health Melbourne VIC Australia; ^16^ Department of Pathology & Cell Biology Columbia University New York NY USA; ^17^ Canadian Blood Services Vancouver BC Canada; ^18^ Department of Pathology & Laboratory Medicine University of British Columbia Vancouver BC Canada; ^19^ Department Unit Transfusion Medicine Sanquin Blood Supply Foundation Amsterdam The Netherlands; ^20^ Department Haematology Erasmus Medical Center Rotterdam The Netherlands

**Keywords:** convalescent plasma, COVID‐19, gap analysis, patient outcome, SARS‐CoV‐2

## Abstract

**Background and objectives:**

Use of convalescent plasma for coronavirus disease 2019 (COVID‐19) treatment has gained interest worldwide. However, there is lack of evidence on its dosing, safety and effectiveness. Until data from clinical studies are available to provide solid evidence for worldwide applicable guidelines, there is a need to provide guidance to the transfusion community and researchers on this emergent therapeutic option. This paper aims to identify existing key gaps in current knowledge in the clinical application of COVID‐19 convalescent plasma (CCP).

**Materials and methods:**

The International Society of Blood Transfusion (ISBT) initiated a multidisciplinary working group with worldwide representation from all six continents with the aim of reviewing existing practices on CCP use from donor, product and patient perspectives. A subgroup of clinical transfusion professionals was formed to draft a document for CCP clinical application to identify the gaps in knowledge in existing literature.

**Results:**

Gaps in knowledge were identified in the following main domains: study design, patient eligibility, CCP dose, frequency and timing of CCP administration, parameters to assess response to CCP treatment and long‐term outcome, adverse events and CCP application in less‐resourced countries as well as in paediatrics and neonates.

**Conclusion:**

This paper outlines a framework of gaps in the knowledge of clinical deployment of CPP that were identified as being most relevant. Studies to address the identified gaps are required to provide better evidence on the effectiveness and safety of CCP use.

## Introduction

Coronavirus disease 2019 (COVID‐19), caused by the severe acute respiratory syndrome coronavirus 2 (SARS‐CoV‐2), was declared a pandemic by the World Health Organization (WHO) on 11 March 2020 [1]. To date, there are no proven therapies for infected patients. Moreover, no vaccines are available, although many are in rapid development and some may be available soon. Based on the concept of passive immunization, human convalescent plasma (CP) from COVID‐19‐convalescent donors has emerged as an option for prevention and treatment of COVID‐19 considering that it can rapidly be made available and, theoretically, could be used for providing immediate immunity to susceptible individuals through viral neutralization [2]. Other proposed mechanisms of action include antibody‐dependent cellular cytotoxicity and/or phagocytosis [3]. Moreover, the use of CP may provide an immunomodulatory benefit via amelioration of macrophage activation and systemic hyper‐inflammation or ‘cytokine storm’ [4].

The interest in the use of CP in managing COVID‐19‐infected patients is based on the historical use of CP in other viral outbreaks such as measles [5], mumps [6] and influenza [7, 8]. CP was also used in viral epidemics such as Spanish influenza A (H1N1) [9], avian influenza A (H5N1), SARS [10], Middle East Respiratory Syndrome and Ebola disease [3, 11, 12]. A retrospective meta‐analysis concluded that CP from survivors of these diseases may reduce mortality, but should be studied in the context of well‐designed trials due to the lack of high‐quality studies and paucity in the published literature [13]. The early case series published from China on the therapeutic use of COVID‐19 convalescent plasma (CCP) showed a potential role in improving clinical symptoms, decreasing viral load and raising serum neutralizing antibody titres [14, 15]. However, these studies featured important limitations, with implications for the conclusions that can be drawn. The WHO recommended scientific studies to explore the feasibility and medical effectiveness for CCP collection and use, and to establish appropriate regulatory conditions including monitoring and reporting patient outcomes [16].

To deploy CCP therapy, various donor, product and patient‐related conditions should be addressed [3]. Guidance is needed to direct blood centres and transfusion services on collection and manufacture of CCP and to support clinicians developing evidence‐based treatment strategies. Existing gaps in knowledge regarding CCP trials need to be identified to enable developing and defining recommendations on patient eligibility, administration, safety and monitoring of adverse events. The deliverables from this project will facilitate study design and analysis of clinical data to determine CCP efficacy and safety, and outcomes can be used to identify areas that need to be explored when facing similar viral pandemics in the future.

## Materials and methods

The International Society of Blood Transfusion (ISBT) through the Clinical Transfusion Working Party (WP) reached out to the ISBT global network to establish a multidisciplinary working group (WG) to address existing practice and gaps in knowledge on the use of CCP. The WG is comprised of members from all WHO regions, with expertise in blood banking, clinical transfusion medicine, adult and paediatric haematology and virology. Altogether, representatives from the ISBT clinical transfusion WP, the ISBT WP on global blood safety, the ISBT WP on transfusion transmissible infectious diseases, the ISBT WP on Haemovigilance, the Asia‐Pacific Blood Network and AABB (formerly American Association of Blood Banks) were included. During weekly teleconferences (April to May 2020) a series of questions pertaining to donor, product and patient domains were devised. The outcome of this project is published in two separate papers. Here, we summarize the knowledge gaps in content areas pertaining to clinical deployment of CCP, based on existing literature at the time of publication, and input from clinical transfusion professionals. Donor‐ and product‐related issues are addressed in a separate paper.

## Results

### Trial and study design

Randomized controlled trials (RCTs) with careful study designs and appropriate control group(s) are preferable. The comparative arm may include standard care, or another intervention, such as non‐convalescent plasma or crystalloid fluid. RCTs will provide the most robust data, but a range of practical considerations will influence study designs possible during the outbreak. Double‐blinded studies may be difficult to conduct in a pandemic situation when it is important to test new treatments as rapidly as possible. To date, there is no obvious placebo comparator to CCP, and non‐convalescent plasma (that does not contain specific anti‐SARS‐CoV‐2 antibodies) could lead to known transfusion‐associated adverse events such as transfusion‐associated circulatory overload (TACO), transfusion‐related acute lung injury (TRALI), allergies or transfusion‐transmitted infections (TTIs). Instead of applying a placebo, a way to decrease bias within the trials is to perform large pragmatic trials that use objective measures of effectiveness, such as assessing a decrease in all‐cause mortality. Platform trials with adaptive design where more than one ‘domain’ may be active concurrently and, where patients can be allocated quickly to promising therapies, may also be efficient ways to conduct high‐quality studies.

Considering that setting up RCTs may not be feasible in all medical settings, other study designs can be employed including cohort studies, case‐control studies and observational studies such as using registries [17]. These studies may provide information that could still be of great value to assess other perspectives, such as the feasibility of collecting, processing and administration of CCP at pre‐defined doses and treatment time‐points in different clinical settings. The choice of study design may be dictated by country‐specific challenges such as feasibility of conducting RCTs and cultural acceptability of enrolment in clinical research in the setting of a pandemic threat.

### Patient eligibility

COVID‐19 convalescent plasma should only be offered as therapy to patients with a laboratory‐confirmed COVID‐19 diagnosis or as prophylaxis in well‐monitored clinical trials exploring prevention of COVID‐19 infection in high‐risk populations. The patient cohorts studied in most of the current therapeutic clinical trials consist of adults with moderate or severe respiratory disease. Eligibility criteria vary substantially, ranging from patients who do not require hospital admission [18] to patients with severe COVID‐19 disease requiring mechanical ventilation [19, 20]. CCP use may have its greatest benefit in patients early in their disease and prior to being placed on mechanical ventilation; however, studies are lacking to make any definitive conclusions [10]. Multiple studies exclude pregnant or breast‐feeding women or patients with co‐morbidities such as renal or cardiac disease [20, 21]. Compassionate use of CCP has been started in settings outside clinical trials for patients with serious or life‐threatening COVID‐19 disease, who are not eligible or who are unable to participate in RCTs [22]. Prophylaxis trials of non‐infected, but at risk subjects including these with history of exposure, are also being designed and now open for inclusion [23, 24].

Considering that the COVID‐19 pandemic is globally dynamic, enrolling patients in clinical trials can be a challenge in countries that are at the acute stage of the pandemic. Obtaining ethical and/or regulatory approvals can delay initiation of the trials. In addition, it becomes a challenge to meet the enrolment target in late stages of the pandemic when the number of eligible cases is decreasing significantly. Collaborative international trials are underway and will be useful in such circumstances [25]. Decision to initiate a CCP trial should be made early in a pandemic after assessing feasibility, benefits and risks. Although blood products are now very safe in most countries with the existing testing for TTIs, recovered COVID‐19‐infected patients who have received CCP manufactured without pathogen reduction treatment (PRT) may be deferred from subsequent CCP collection. In addition, CCP donors may be eligible to receive allogeneic CCP in case of COVID‐19 re‐infection or reactivation.

### COVID‐19 convalescent plasma dose, frequency and timing of administration

COVID‐19 convalescent plasma can be collected by apheresis or whole blood donation. Apheresis yields 200–800 ml of CCP that can be divided into 1–4 separate units before freezing. There is a wide variation in the CCP dose used [26] due to a lack of standardization in study design, variation in CCP collection methods, production and administration. In early studies from China, two consecutive transfusions of 200–250 ml of ABO‐compatible CCP were used in one study [14], whereas a single 200 ml dose with anti‐SARS‐CoV‐2 titre >1:640 was tested in another study [27]. A dose of 200 ml, followed by 1–2 doses of 200 ml according to disease severity and patient tolerance, has been recommended by some authors [28]. This was also the case for CP use in other viral outbreaks, including H5N1 [8].

At this stage, there is no evidence for which dose and timing is best to optimize patient outcome [26]. A variety of CCP doses is currently under evaluation in registered clinical trials ranging between 200 and 600 ml per adult patient, or defined according to the patient's body weight or by the number of units to be transfused [29]. The minimal effective dose, and whether that is related to a specific neutralizing antibody titre, is currently unknown. Because viraemia peaks in the first week of infection in most viral illnesses, and because the primary immune response typically develops by day 10–14, followed by viral clearance, administering CCP early in the disease course could theoretically be more effective [10]. Based on the experience in other viral infections, CP should be used early in the disease before the inflammatory syndrome starts and the peak of production of endogenous IgM and IgG antibodies [8, 30]. In early studies of SARS patients, better outcomes were seen in those given CCP before day 14, as compared to later time‐points [10]. This approach would be expected to be most effective in COVID‐19‐infected patients; however, it has not yet been shown in clinical trials. Early administration for COVID‐19‐infected patients is also believed to prevent innate immune cell migration and avoid lung damage [4].

Whether additional transfusions should be given, and when, is also currently unknown. Factors to be analysed include transfused volume, response to treatment, and the risk of adverse events. Patient clinical and laboratory criteria that may determine the need of an additional dose and the timing of its administration from the first dose are yet to be defined.

### Parameters to assess response and outcome

The safety and efficacy of CCP transfusion are not yet established and new data on patient outcomes are emerging continuously [27, 31, 32, 33]. Previous meta‐analyses on CP use in other viral infections reported improvement in clinical signs and symptoms, hospital length of stay, viral load and mortality [8, 13]. One meta‐analysis identified the potential for CP to reduce mortality in severe acute respiratory infections of other aetiologies including SARS‐CoV‐1 and H1N1 influenza [13]. Outcomes included mortality, hospital length of stay, requirement for and duration of critical care support, viral antibody level, viral load and adverse events. However, the same meta‐analysis reported methodological heterogeneity and moderate‐high risk of bias in the studies conducted.

Limited early data on CCP use suggest clinical benefit with reductions in body temperature, improved Sequential Organ Failure Assessment (SOFA) score, less need of respiratory support, improved lymphocyte count and inflammatory markers, increases in IgG, IgM and neutralizing antibody titres, and reduction in viral load [14, 27]. Another study reported reduced pulmonary lesions, by chest CT scans, after CCP transfusion [27]. Use of globally accepted objective disease severity definitions and mobility end‐points when assessing response to CCP is preferred to enable comparison between studies [34]. The European Commission recommended that hospitals report various parameters, including clinical symptoms, laboratory results, a disease progression scale, length of hospitalization, and serious adverse events [35]. A publicly accessible database is set up to gather outcome data and to allow for meta‐analyses to evaluate safety and efficacy on a regular basis [17]. It is paramount to report end‐point results of such studies regardless of their study design to enable acquisition of data and information on the feasibility of CCP use, its effectiveness and safety.

The authors acknowledge the need for RCTs to evaluate CCP use objectively among different patient populations. It is important to assess feasibility and efficacy under standard regulatory conditions, particularly regarding ethical conduct, appropriate CCP collection, and the monitoring and reporting of patient responses and outcomes [16]. Assessing the impact of other confounders, such as patient co‐morbidities, timing of administration and effects of other treatments (e.g., anti‐viral drugs and cytokine inhibitors), necessitates stringent evaluation based on pre‐defined clinical signs and symptoms and laboratory parameters (e.g., inflammatory markers, serum cytokine and viral antibody levels, viral load). Other outcomes include mortality, hospital length of stay, duration of critical care support (e.g., days on a ventilator and/or in an intensive care unit), severe adverse events and treatment complications.

Comparing clinical and laboratory patient responses to CCP antibody characteristics may identify patient and donor factors that predict clinical efficacy. Access to reliable COVID‐19 antibody testing varies, and is rapidly evolving, affecting the ability to qualify CCP donors and characterize their donations. Prior to implementation of COVID‐19 antibody assays with titre thresholds as release criterium for CCP, clinical trials should consider requesting retention samples from CCP units to be stored until assays are available. This will improve the ability for objectively measuring product characteristics and efficacy and outcomes of CCP infused prior to routine use of these assays.

### Adverse events

No serious adverse events were reported in the systematic review of CP use in other viral infections [13]. In addition, no serious adverse effects were reported from the initially published studies regarding CCP use [27]. That said, from the first Cochrane review of reported case series, the adverse events rates were reported to be very low [26]. Moreover, a study reported a low rate of serious adverse events in the first four hours of transfusion (<1%) [36]. However, under reporting of adverse events cannot be excluded. Risks associated with CCP are likely to be the same as those with standard plasma, including TTIs, mild transfusion reactions (e.g., allergic and febrile) to potentially life‐threatening transfusion reactions (e.g., TACO, TRALI and anaphylaxis/anaphylactoid reactions) [37]. TACO and TRALI are particularly concerning in severe COVID‐19 given the underlying acute lung injury and potential priming of the pulmonary endothelium [38, 39]; this highlights the importance of CCP donor selection to avoid high risk donors. Thus, the European Union program requires CCP donors without a history of blood transfusion and female donors who have never been pregnant, or are tested and found negative for anti‐HLA/HPA/HNA antibodies using a validated assay [35]. Pre‐treatment to minimize transfusion reactions (e.g. acetaminophen and diphenhydramine) may be considered, as needed, or if the patient had previously needed pre‐medication for blood transfusions. Whatever dose of CCP is used, patients at risk of TACO (small stature, low body weight, elderly, known or suspected renal or cardiac dysfunction) should be transfused slowly – at a rate as low as 1 ml/kg/h – and closely monitored throughout the infusion [40].

Reporting adverse events using internationally agreed haemovigilance definitions will assist in comparing results between studies [41, 42, 43]. Donor and patient adverse events need to be reported within institutional and national haemovigilance frameworks using internationally agreed definitions to gather more information on the safety of CCP collection and its use in adult and paediatric patients. Cooperation with international haemovigilance programs is preferable.

There is a theoretical risk of transmitting SARS‐CoV‐2 by transfusion, especially with the current lack of donor screening for common respiratory viruses [38]. In one recent study, four asymptomatic donors, out of 2430 screened platelet and whole blood donations, had detectable SARS‐CoV‐2 RNA in their blood [44]. However, detectable RNA does not necessarily imply infectivity. To the best of our knowledge, there has never been a report of respiratory virus transmission via blood transfusion; nonetheless, this needs to be assessed by ongoing surveillance. Another potential risk is antibody‐dependent enhancement (ADE), whereby antibodies developed during past infection with a different viral serotype exacerbate clinical severity of the current illness. This was seen with dengue virus, among other viral infections [45, 46]. It is hypothesized that the mechanism involves IgG antibody Fc‐region binding to the Fc gamma receptor on an immune cell, such that the Fc gamma receptor functionally mimics the actual viral receptor and, thereby, mediates viral entry [47]. There have been no reports of this phenomenon occurring with the SARS‐CoV‐1 or MERS viruses as a result of CP transfusion. Nonetheless, specific studies to assess this potential risk are required, particularly regarding vaccine design, use of PRT and monoclonal antibody‐based therapy. Finally, there is a theoretical risk that CCP could exacerbate underlying coagulopathy associated with severe COVID‐19 [48]. This was not reported in any of the recently published studies [26, 33, 36]. These potential risks and the fact that, at this stage, there is no specific anti‐SARS‐CoV‐2 treatment available, should be discussed with the patient at time of enrolment in CCP clinical trials.

### Application in paediatric and neonatal medicine

Early data suggest that paediatric COVID‐19 cases might experience different symptoms than adults with children overall showing less severe disease than adults [49]. Based on all reports so far, less than 10% of children had severe or critical disease and mortality was rare [49]. Children with chronic lung and/or cardiovascular disease or immunodeficiency or on immunosuppression may be at higher risk for worse outcomes [50, 51, 52]. There is a scarcity of data specifically on CP use in paediatric and neonatal populations. During the Spanish flu, paediatric single doses of 50 ml of CP were attempted but no clear paediatric‐specific outcomes are listed [8]. Given the dearth of other treatment options, CP can potentially play a key role and be a safe and efficacious treatment modality in children and neonates, which if instituted in a timely manner may reduce progression from mild to more severe disease. But this will need extremely careful evaluation in well‐planned clinical trials and prospective studies.

Very few trials on use of CPP currently include children or neonates; however, few trials are planning to include children [24, 53, 54]. In the United States, a study aims to evaluate the safety and pharmacokinetics of human CCP in high‐risk children (1 month–18 years), either with confirmed infection or with high risk exposure. CCP, with anti‐SARS‐CoV‐2 antibody titres ≥1:320, at a dose of 5 ml/kg, with a maximum volume of 500 ml, will be used [24]. In Canada, a randomised, multi‐centred, open‐label Phase 2 clinical trial of the safety and efficacy of CCP for treatment of COVID‐19 disease in hospitalised children has been launched [53]. This protocol allows a 10 ml/kg dose up to a maximum dose of 500 ml vs. standard of care as the control arm. It is challenging to perform clinical trials in this age group due to lower patient numbers. Therefore, the importance of multi‐institutional collaborative national and international efforts cannot be overemphasized. Multi‐institute registries collating observational data on CCP use and outcomes can be very important in the interim, while trial results are awaited.

Kawasaki‐like inflammatory syndromes (Multisystem Inflammatory Syndrome in Children; MIS‐C) have been reported in children with COVID‐19 [55]. Most reports, both anecdotal and published, described the MIS‐C occurring after infection with SARS‐CoV‐2. However, some report that the virus can be detected concurrently with MIS‐C [56]. Whether affected children may benefit from CCP remains unclear and no recommendation can be made with certainty. If the patient is actively infected with SARS‐CoV‐2 at time of MIS‐C then treatment with CCP might be beneficial. More studies are required to establish safety and efficacy of CCP in this syndrome.

### Less‐resourced countries

Resource constraints at both micro‐ and macro‐level impact the provision of healthcare infrastructure as well as the ability to access this limited healthcare. The net effect is late presentation of significantly ill patients who compete for limited resources with other patients, often displacing such patients and further depleting the available resources. In addition, poor socio‐economic circumstances contribute to rapid spread of disease, high rates of co‐morbidities, all of which may contribute to poor patient outcomes, especially given limited availability of critical care facilities. At high rates of community outbreaks, however, especially once healthcare resources are saturated, higher resourced countries see similar issues.

There are many challenges faced by medical systems in low‐ and middle‐income countries (LMICs) that may limit enrolling patients in CCP treatment programmes. Performing robust clinical trials against this background is problematic, as is borne out by the work on CP use during the previous Ebola crisis [57, 58] leading to the potential empirical or observational use of CP in these settings. All possible attempts should be made to confirm safety and efficacy prior to redirecting limited resource to large‐scale collection and provision of CCP. It is important to avoid empirical use of CCP based on symptomatology; particularly because COVID‐19 has a wide range of presenting symptoms with significant overlap with other communicable diseases. Where feasible, CCP use should ideally be part of clinical trials, even if limited in scope, with clear, preferably clinical end‐points, which do not require sophisticated laboratory investigations. However, plasma infusions in these setting are not without risk; high levels of communicable diseases and limited access to robust testing systems, poses particular risks in these settings, necessitating detailed risk‐benefit assessments. Supply from other countries across international borders is impeded by many regulatory, financial and logistic barriers and challenges.

### Ethical consideration

The adoption of CCP for treatment of COVID‐19 has introduced a number of ethical challenges. Foremost, it remains an unproven therapy, despite a growing literature suggesting that it may be beneficial. Indeed, CCP was adopted quickly and widely in the absence of strong evidence of benefit, instead relying on case reports and uncontrolled observational studies to support its use [14, 15]. Those data suffer from serious methodologic limitations, not least of which is the potential for confounding in late‐stage disease. As one example, most of the patients who have received CCP have also been subjected to a range of other therapies. Second, recent data have afforded insight into the safety of use, suggesting that the risk of CCP is comparable to that of standard plasma [36]. Nonetheless, early in the pandemic when treatment options were otherwise minimal, transfusion of CCP was undertaken before safety data was available to specifically address the theoretical risks of SARS‐CoV‐2 transmission, ADE and exacerbation of underlying coagulopathy [38].

The demand for CCP continues to increase as it gains media attention as a viable treatment due to its anecdotal successes, its relative ease to manufacture from recovered patients, and as other therapeutic modalities fail to show benefit in clinical trials. There is insufficient inventory to support all patients in the notable absence of robust clinical data. While dual inventories could avoid competition between the clinical trials and compassionate use of CCP, there is more likely to be a single source to draw from. Compassionate CCP use may also impede enrolment into clinical trials, particularly if the study design includes randomisation offering the potential of placebo rather than CCP.

From an ethical perspective, all patients – including those enrolled in RCTs – should receive the best available supportive care as soon as appropriate and available. Considering the lack of high‐level evidence to support the use of CCP at this time, the option to allow patients to cross over to the ‘treatment group’ who were initially randomized into the control group, and who show progressive disease after the primary end‐point of the trial has been reached, can be considered. The latter may not only be an ethical option but could also allow data acquisition on an additional CCP infusion time‐point in a given study after assessment of the primary end‐points.

A broader question relates to the ethics of implementation of an unproven therapy in LMICs, the majority of which are unable to meet transfusion demand given a myriad of systemic challenges [59, 60], but which may also have extremely limited access to other therapies. CCP may further strain those transfusion services. Using whole blood collection techniques could decrease the overall safety of the local blood supply by collecting (largely) first time donors, who traditionally have higher rates of TTIs. Diverting limited healthcare and blood establishment resources to the collection and provision of products with limited evidence of efficacy within a particular setting may more broadly negatively affect public health delivery.

## Conclusions

We identified key questions and gaps in knowledge pertaining to the clinical use of CCP, including in special settings for paediatric and neonatal patients, as well as in less‐resourced countries, and we suggest points to consider for developing new trials (Table 1). Acknowledging the substantial heterogeneity of the clinical CCP landscape the medical field has to deal with, this gap paper could help to put some studies into context, and it could contribute to conduct more streamlined and co‐ordinated future studies. Central questions to be clarified are whether CCP is safe and effective for adult and paediatric patients with COVID‐19. A recent Cochrane Systematic Review has shown no evidence to support CCP use based on the very limited existing reports [26]. However, substantial amounts of data are being published every day. Thus, addressing gaps in knowledge identified in this document, together with emerging evidence, is expected to identify the benefits and risks of CCP, thereby providing a robust basis for defining its future therapeutic use.

**Table 1:** Identified knowledge gaps and points for consideration in the use of CCP for treating COVID‐19 patients

Objective	Identified gaps	Points to consider
Trial and study design	What is the best way to assess effectives and safety of CCP? What control arm treatment should be employed in designing randomized clinical trials? What other treatment alternatives to CCP are available? What supportive measures will be available in the inclusion arm?	Wherever possible, use of CCP should be within the context of a clinical trial until or unless its efficacy and safety are established. If CCP is used outside a clinical trial, data should still be collected to gather experience and outcomes. Effectiveness of CPP compared to other treatment alternatives should be assessed whenever possible. All patients should receive the best available supportive care as soon as being appropriate and available.
Patient eligibility	Who would benefit most from CCP treatment? Who would benefit from CCP prophylaxis? What is the role of compassionate use of CCP, if any, outside a clinical trial? Could CCP be not beneficial or even harmful?	Clinical trials should: Define the disease settings to assess which patients will benefit the most from therapeutic and prophylactic CCP and/or when CCP is not beneficial or even harmful. Define patient eligibility criteria if any for compassionate use of CCP.
CCP dose, frequency and timing of administration	What is the minimal acceptable CCP dose to be effective? What is the optimal dose of CCP? Does CCP dose vary between clinical settings (e.g. disease severity, different patient groups)? When should CCP be administered in the course of the disease? What clinical criteria define the need for a repeat dose(s)?	Clinical trials should: Define minimum CCP dose needed for efficacious treatment. Assess optimum CCP dose in a range of disease severities and clinical settings. Define appropriate time‐points for CCP administration in the course of the disease for efficacious treatment. Define clinical criteria that allow (repeated) CCP administration.
Parameter to assess response and outcome	What clinical and laboratory parameters should be used to monitor response? What are the best clinical outcomes to measure and what morbidity end‐points should be assessed? How outcomes are related to antibody characteristics and titre levels? What confounders could impact patients’ outcomes?	Use of routinely collected data as much as possible to reduce workload pressure on front‐line staff caring for patients. Use precisely defined and globally accepted objective disease severity definitions, morbidity and quality of life end‐points to assess clinical impact of CCP transfusions and to allow comparison of studies. Assess for CCP antibody characteristics and titre levels, and compare these with the laboratory and clinical response. Assess and control for confounders that could impact patients' outcomes.
Adverse events	Is CCP use safe? When is CCP uniquely unsafe? Is CCP transfusion associated with higher risks of adverse events compared to standard plasma? What hemovigilance definitions should be used to characterized adverse events in transfused patients? Can SARS‐CoV‐2 be transmitted by blood transfusion? Can CCP transfusion induce ADE or exacerbate underlying coagulopathy? Is pathogen reduction technology warranted to reduce TTI risk? Are there any novel adverse events that occur with CCP?	Monitor safety of CCP and define settings in which it should not be used. Monitor patients for adverse events while on treatment. Use internationally agreed definitions to gather more information on the safety of CCP collection and its use. Monitor whether SARS‐CoV‐2 can be transmitted through blood and blood products. Monitor whether CCP transfusion can induce ADE or exacerbate underlying coagulopathy. Determine if use of pathogen reduction technology is warranted. Assess for any SURARs that may occur with CCP use.
Application in paediatric and neonatal medicine	What would be the eligibility criteria for use of CCP in paediatrics and neonates? What CCP dose to be used and how frequent? What clinical outcome should be assessed?	Clinical trials should: Define eligibility criteria for use of CCP in paediatrics and neonates. Define CCP dose and frequency of administration. Define paediatric‐specific clinical outcome measures to be assessed, especially outcome measures that can be objectively measured.
Less‐resourced countries	How to determine if use of CCP is feasible in settings of limited resources? Are there any international programs to facilitate access to CCP for patients in medical systems with limited resources?	Perform risk assessments for the use of CCP ideally including a review of the safety of the country's blood supply in general and within the context of individual facilities in particular. Develop (international) programs to facilitate access to CCP for patients in medical systems with limited resources.
Ethical considerations	How to prioritize CCP use if limited supply or if competition exists on the existing inventory between clinical trials and compassionate use? When to consider a cross‐over of patients from the control arm to the treatment arm in CCP clinical trials? How to implement CCP in settings with challenges in providing sufficient blood supply in LMICs?	Define a mechanism on how to meet demand with insufficient CCP supply. In the absence of current effective treatment options, consideration should be given to patients in control arms crossing over to CCP treatment arms in case of disease progression. Diversion of resources away from routine blood collections in LMICs need to be carefully assessed.

COVID‐19, Coronavirus disease 2019; CCP, COVID‐19 convalescent plasma; LMICs, Low‐ and middle‐income countries; SARS‐CoV‐2, Severe acute respiratory syndrome coronavirus 2; SURARs, Suspected unexpected serious adverse reactions; TACO, Transfusion‐associated circulatory overload; TRALI; Transfusion‐related acute lung injury; TTI, Transfusion‐transmitted infection.

## Conflict of interests

Evan M. Bloch reports personal fees and non‐financial support from Terumo BCT, personal fees and non‐financial support from Grifols Diagnostics Solutions, outside of the submitted work; EMB is a member of the United States Food and Drug Administration (FDA) Blood Products Advisory Committee. Any views or opinions that are expressed in this manuscript are that of the author's, based on his own scientific expertise and professional judgment; they do not necessarily represent the views of either the Blood Products Advisory Committee or the formal position of FDA and also do not bind or otherwise obligate or commit either Advisory Committee or the Agency to the views expressed. Dana Devine a member of the scientific advisory board of Macopharma and received research support from TerumoBCT, Hemanext and Macopharma.

## Funding

Evan M. Bloch effort is supported in part by the National Heart Lung and Blood Institute (1K23HL151826‐01).

## References

[1] World Health Organization : Coronavirus disease (COVID‐19) outbreak. https://www.who.int/westernpacific/emergencies/covid‐19. Published 2020. [Last accessed 1st May, 2020]

[2] Zhang JS , Chen JT , Liu YX , *et al*: A serological survey on neutralizing antibody titer of SARS convalescent sera. J Med Virol 2005; 77:147–150 16121363 10.1002/jmv.20431 PMC7167078

[3] Casadevall A , Pirofski L‐A : The convalescent sera option for containing COVID‐19. J Clin Investig 2020; 130:1545–1548 32167489 10.1172/JCI138003 PMC7108922

[4] Rojas M , Rodríguez Y , Monsalve DM , *et al*: Convalescent plasma in Covid‐19: possible mechanisms of action. Autoimmun Rev 2020; 19:102554 32380316 10.1016/j.autrev.2020.102554 PMC7198427

[5] Gallagher JR : Use of convalescent measles serum to control measles in a preparatory school. Am J Public Health Nations Health. 1935; 25:595–598 18014217 10.2105/ajph.25.5.595 PMC1559172

[6] Rambar AC : Mumps: use of convalescent serum in the treatment and prophylaxis of orchitis. Am J Dis Child 1946; 71:1–13 21018124

[7] Luke TC , Casadevall A , Watowich SJ , *et al*: Hark back: passive immunotherapy for influenza and other serious infections. Crit Care Med 2010; 38:e66–e73 20154602 10.1097/CCM.0b013e3181d44c1e

[8] Luke TC , Kilbane EM , Jackson JL , *et al*: Meta‐analysis: convalescent blood products for Spanish influenza pneumonia: a future H5N1 treatment? Ann Intern Med 2006; 145:599–609 16940336 10.7326/0003-4819-145-8-200610170-00139

[9] Hung IF , To KK , Lee C‐K , *et al*: Convalescent plasma treatment reduced mortality in patients with severe pandemic influenza A (H1N1) 2009 virus infection. Clin Infect Dis 2011; 52:447–456 21248066 10.1093/cid/ciq106 PMC7531589

[10] Cheng Y , Wong R , Soo Y , *et al*: Use of convalescent plasma therapy in SARS patients in Hong Kong. Eur J Clin Microbiol Infect Dis 2005; 24:44–46 15616839 10.1007/s10096-004-1271-9 PMC7088355

[11] Sahr F , Ansumana R , Massaquoi T , *et al*: Evaluation of convalescent whole blood for treating Ebola Virus Disease in Freetown. Sierra Leone. Journal of Infection. 2017; 74:302–309 10.1016/j.jinf.2016.11.009 PMC7112610 27867062

[12] Casadevall A , Scharff MD : Return to the past: the case for antibody‐based therapies in infectious diseases. Clin Infect Dis 1995; 21:150–161 7578724 10.1093/clinids/21.1.150 PMC7197598

[13] Mair‐Jenkins J , Saavedra‐Campos M , Baillie JK , *et al*: The effectiveness of convalescent plasma and hyperimmune immunoglobulin for the treatment of severe acute respiratory infections of viral etiology: a systematic review and exploratory meta‐analysis. J Infect Dis 2015; 211:80–90 25030060 10.1093/infdis/jiu396 PMC4264590

[14] Shen C , Wang Z , Zhao F , *et al*: Treatment of 5 critically ill patients with COVID‐19 with convalescent plasma. JAMA 2020; 323:1582 32219428 10.1001/jama.2020.4783 PMC7101507

[15] Duan K , Liu B , Li C , *et al* Effectiveness of convalescent plasma therapy in severe COVID‐19 patients. Proceedings of the National Academy of Sciences 2020; 117(17):9490–9496 10.1073/pnas.2004168117 PMC7196837 32253318

[16] WHO Blood Regulators Network : Interim position paper on blood regulatory response to the evolving out‐break of the 2019 novel coronavirus SARS‐CoV‐2*. In: 2020

[17] EU CCP Database : Covid‐19 convalescent plasma collection and transfusion in the EU. https://ec.europa.eu/health/blood_tissues_organs/covid‐19_en. Published 2020. [Last accessed 15th May, 2020]

[18] Convalescent plasma vs. placebo in emergency room patients with COVID‐19. https://clinicaltrials.gov/ct2/show/NCT04355767?term=NCT04355767&draw=2&rank=1. Published 2020. [Last accessed 11th May, 2020]

[19] Convalescent plasma in ICU patients with COVID‐ 19‐induced Respiratory Failure. https://clinicaltrials.gov/ct2/show/NCT04353206?term=convalescent+plasma&cond=COVID&draw=2&rank=10. Published 2020. [Last accessed 2nd May, 2020]

[20] A randomized, prospective, open label clinical trial on the use of convalescent plasma compared to best supportive care in patients with severe COVID‐19. https://www.clinicaltrialsregister.eu/ctr‐search/trial/2020‐001310‐38/DE#A. Published 2020. [Last accessed 2nd May, 2020]

[21] Convalescent Plasma for Patients With COVID‐19: a randomized, open label, parallel, controlled clinical study (CP‐COVID‐19). https://clinicaltrials.gov/ct2/show/NCT04332835?term=NCT04332835&draw=2&rank=1. Published 2020. [Last accessed 11th May, 2020]

[22] Expanded access to convalescent plasma for the treatment of patients with COVID‐19. https://clinicaltrials.gov/ct2/show/NCT04338360. Published 2020. [Last accessed 6th May, 2020]

[23] Efficacy and safety human coronavirus immune plasma (HCIP) vs. control (SARS‐CoV‐2 Non‐immune Plasma ) among adults exposed to COVID‐19 (CSSC‐001). https://clinicaltrials.gov/ct2/show/NCT04323800?term=john+hopkins&cond=convalescent+plasma&draw=2&rank=4. Published 2020. [Last accessed 9th May, 2020]

[24] National COVID‐19 Convalescent Plasma Project : Protocols for pediatrics. https://ccpp19.org/healthcare_providers/component_3/protocols_for_pediatrics.html. Published 2020. [Last accessed 26th April, 2020]

[25] A randomised, embedded, multi‐factorial, adaptive platform trial for community‐acquired pneumonia. https://www.remapcap.org/. Published 2020. [Last accessed 10th June, 2020]

[26] Valk SJ , Piechotta V , Chai KL , *et al*: Convalescent plasma or hyperimmune immunoglobulin for people with COVID‐ 19: a rapid review. Cochrane Database Syst Rev 2020; 5:CD013600 10.1002/14651858.CD013600 PMC7271896 32406927

[27] Duan K , Liu B , Li C , *et al*: Effectiveness of convalescent plasma therapy in severe COVID‐19 patients. Proc Natl Acad Sci 2020; 117:202004168 10.1073/pnas.2004168117 PMC7196837 32253318

[28] Epstein J , Burnouf T : Points to consider in the preparation and transfusion of COVID‐19 convalescent plasma. Vox Sang 2021; 116:13–4 32319102 10.1111/vox.12939 PMC7264781

[29] US National Library of Medicine : Clinical Trials. https://clinicaltrials.gov/ct2/results?cond=COVID‐19&term=convalescent&cntry=&state=&city=&dist=&Search=Search. [Last accessed 26th April, 2020]

[30] Soo Y , Cheng Y , Wong R , *et al*: Retrospective comparison of convalescent plasma with continuing high‐dose methylprednisolone treatment in SARS patients. Clin Microbiol Infect 2004; 10:676–678 15214887 10.1111/j.1469-0691.2004.00956.x PMC7129386

[31] Liu ST , Lin H‐M , Baine I , *et al* Convalescent plasma treatment of severe COVID‐19: a matched control study. medRxiv. 2020 10.1038/s41591-020-1088-9 32934372

[32] Li L , Zhang W , Hu Y , *et al*: Effect of convalescent plasma therapy on time to clinical improvement in patients with severe and life‐threatening COVID‐19: a randomized clinical trial. JAMA 2020:e2010044 10.1001/jama.2020.10044. [Online ahead of print] PMC7270883 32492084

[33] Salazar E , Perez KK , Ashraf M , *et al*: Treatment of Coronavirus Disease 2019 (COVID‐19) Patients with Convalescent Plasma. Am J Pathol. 2020 10.1016/j.ajpath.2020.05.014. [Online ahead of print] PMC7251400 32473109

[34] Core outcome set developers' response to COVID‐19 (15th April 2020). http://www.comet‐initiative.org/Studies/Details/1538. Published 2020. [Last accessed 11th May, 2020]

[35] An EU programme of COVID‐19 convalescent plasma collection and transfusion. In. Guidance on collection, testing, processing, storage, distribution and monitored use. 1.0 ed. Brussels European Commission; Directorate‐General for Health and Food Safety 2020:7

[36] Joyner M , Wright RS , Fairweather D , *et al* Early safety indicators of COVID‐19 convalescent plasma in 5,000 patients. J Clin Invest. 2020 10.1172/JCI140200. [Online ahead of print] PMC7456238 32525844

[37] Leider JP , Brunker PA , Ness PM : Convalescent transfusion for pandemic influenza: preparing blood banks for a new plasma product? Transfusion 2010; 50:1384–1398 20158681 10.1111/j.1537-2995.2010.02590.x PMC7201862

[38] Bloch EM , Shoham S , Casadevall A , *et al*: Deployment of convalescent plasma for the prevention and treatment of COVID‐19. J Clin Investig 2020; 130:2757–2765 32254064 10.1172/JCI138745 PMC7259988

[39] Tiberghien P , de Lambalerie X , Morel P , *et al* Collecting and evaluating convalescent plasma for COVID‐19 treatment: why and how? Vox Sang 2020 10.1111/vox.12926. [Online ahead of print] 32240545

[40] Fung MK , Grossman BJ , Hillyer CD , Westhoff CM : Technical Manual. 19th edn. Bethesda, MD: AABB; 2017:498

[41] ISBT : Proposed standard definitions for surveillance of non infectious adverse transfusion reactions. https://www.isbtweb.org/fileadmin/user_upload/Proposed_definitions_2011_surveillance_non_infectious_adverse_reactions_haemovigilance_incl_TRALI_correction_2013_TACO_correction_2018.pdf. Published 2013. [Last accessed 17th May, 2020]

[42] Vlaar AP , Toy P , Fung M , *et al*: A consensus redefinition of transfusion‐related acute lung injury. Transfusion 2019; 59:2465–2476 30993745 10.1111/trf.15311 PMC6850655

[43] Transfusion‐associated circulatory overload (TACO) Definition. 2018 http://www.isbtweb.org/fileadmin/user_upload/TACO_2018_definition_March_2019.pdf

[44] Chang L , Zhao L , Gong H , Wang L : Severe acute respiratory syndrome coronavirus 2 RNA detected in blood donations. Emerg Infect Dis 2020; 26:1631–1633 32243255 10.3201/eid2607.200839 PMC7323524

[45] Dejnirattisai W , Supasa P , Wongwiwat W , *et al*: Dengue virus sero‐cross‐reactivity drives antibody‐dependent enhancement of infection with zika virus. Nat Immunol 2016; 17:1102–1108 27339099 10.1038/ni.3515 PMC4994874

[46] Guzman MG , Alvarez M , Halstead SB : Secondary infection as a risk factor for dengue hemorrhagic fever/dengue shock syndrome: an historical perspective and role of antibody‐dependent enhancement of infection. Adv Virol 2013; 158:1445–1459 10.1007/s00705-013-1645-3 23471635

[47] Wan Y , Shang J , Sun S , *et al*: Molecular mechanism for antibody‐dependent enhancement of coronavirus entry. J Virol 2020; 94:e02015 31826992 10.1128/JVI.02015-19 PMC7022351

[48] Giannis D , Ziogas IA , Gianni P : Coagulation disorders in coronavirus infected patients: COVID‐19, SARS‐CoV‐1, MERS‐CoV and lessons from the past. J Clin Virol 2020; 127:104362 32305883 10.1016/j.jcv.2020.104362 PMC7195278

[49] Dong Y , Mo X , Hu Y , *et al*: Epidemiology of COVID‐19 among children in China. Pediatrics 2020; 145:e20200702 32179660 10.1542/peds.2020-0702

[50] Huijing Ma JH , Tian Jie , Zhou Xi *et al*: A single‐center, retrospective study of COVID‐19 features in children: a descriptive investigation. BMC Med. 2020; 18:123 32370747 10.1186/s12916-020-01596-9 PMC7200209

[51] CDC COVID‐19 Response Team , Bialek S : Coronavirus Disease 2019 in Children—United States, February 12–April 2, 2020. MMWR Morb Mortal Wkly Rep 2020; 69:422–426 32271728 10.15585/mmwr.mm6914e4 PMC7147903

[52] Parri N , Lenge M , Buonsenso D : Children with Covid‐19 in pediatric emergency departments in Italy. N Engl J Med 2020 10.1056/NEJMc2007617. [Online ahead of print] PMC7206930 32356945

[53] Efficacy of human Coronavirus‐immune convalescent plasma for the treatment of COVID‐19 disease in hospitalized children (CONCOR‐KIDS). https://clinicaltrials.gov/ct2/show/NCT04377568. Published 2020. [Last accessed 11th May, 2020]

[54] A randomised trial of treatments to prevent death in patients hospitalised with COVID‐19 (coronavirus). http://www.isrctn.com/ISRCTN50189673. Published 2020. [Last accessed 11th May, 2020]

[55] Viner RM , Whittaker E : Kawasaki‐like disease: emerging complication during the COVID‐19 pandemic. Lancet 2020; 395:1741–1743 32410759 10.1016/S0140-6736(20)31129-6 PMC7220168

[56] Chiotos K , Bassiri H , Behrens EM , *et al*: Multisystem inflammatory syndrome in children during the COVID‐19 pandemic: a case series. J Pediatr Infect Dis Soc 2020 10.1093/jpids/piaa069. [Online ahead of print] PMC7313950 32463092

[57] Van Griensven J , Edwards T , de Lamballerie X , *et al*: Evaluation of convalescent plasma for Ebola virus disease in Guinea. N Engl J Med 2016; 374:33–42 26735992 10.1056/NEJMoa1511812 PMC5856332

[58] Edwards T , Semple MG , De Weggheleire A , *et al*: Design and analysis considerations in the Ebola_Tx trial evaluating convalescent plasma in the treatment of Ebola virus disease in Guinea during the 2014–2015 outbreak. Clin Trials 2016; 13:13–21 26768570 10.1177/1740774515621056 PMC4738238

[59] Weimer A , Tagny C , Tapko J , *et al*: Blood transfusion safety in sub‐Saharan Africa: a literature review of changes and challenges in the 21st century. Transfusion 2019; 59:412–427 30615810 10.1111/trf.14949

[60] Roberts N , James S , Delaney M , *et al*: The global need and availability of blood products: a modelling study. Lancet Haematol 2019; 6:e606–e615 31631023 10.1016/S2352-3026(19)30200-5

